# Health Locus of Control and Physical Activity in Liver Transplant Recipients

**DOI:** 10.7759/cureus.65817

**Published:** 2024-07-31

**Authors:** Meriç Yıldırım, Burcin Aktar, Hatice Nihan Bozkurt, Tufan Egeli, Tarkan Unek

**Affiliations:** 1 Faculty of Physical Therapy and Rehabilitation, Dokuz Eylul University, Izmir, TUR; 2 Institute of Health Sciences, Dokuz Eylul University, Izmir, TUR; 3 Hepatopancreaticobiliary Surgery and Liver Transplantation Unit, Department of General Surgery, Faculty of Medicine, Dokuz Eylul University, Izmir, TUR

**Keywords:** doctors locus of control, internal locus of control, locus of control, physical activity, liver transplantation

## Abstract

Introduction: Health locus of control has the potential to influence health behavior among individuals with chronic diseases. However, little is known regarding the relationship between locus of control and physical activity in transplant recipients. The aim of the study was to investigate health locus of control and physical activity in liver transplant recipients.

Methods: Seventy-six liver transplant recipients were enrolled. The Multidimensional Health Locus of Control - Form C, including internal, chance, doctors, and powerful others subdimensions, was used to evaluate the health locus of control. Physical activity was assessed with the International Physical Activity Questionnaire - Short Form.

Results: The median internal health locus of control score was 28.54. Thirty-six participants had low, 35 participants had moderate, and five participants had high physical activity. Internal health locus of control was higher in patients having moderate activity compared to patients with low activity (p=0.04). "Doctors" subdimension of health locus of control was negatively correlated with walking (r=-0.38, p=0.02) and total activity scores among patients with low activity (r=-0.37, p=0.02).

Conclusion: Liver transplant recipients with moderate activity present higher internal health locus of control scores, suggesting a possible influence of physically active behavior on the beliefs of one’s own control over disease management. As the activity level increases, the belief that health outcomes are largely the responsibility of health professionals decreases among liver transplant recipients with low activity. Promoting physical activity will help develop the control of one's own actions on health after liver transplantation.

## Introduction

Solid organ transplantation is a milestone in a recipient’s life [1]. In spite of being the gold standard surgical procedure for the treatment of end-stage liver diseases, liver transplantation, similar to other solid organ transplantations, can be demanding for recipients and their caregivers [2]. After the transplantation procedure, patients are expected to comply with strict rules regarding adherence to pharmacological treatment, specifically immunosuppressive medications, adapting their lifestyles to the new situation, developing health behaviors including regular physical activity/exercise, healthy diet, avoidance of alcohol/smoking, avoiding risks for postoperative complications and coping with the physical and psychosocial consequences of the condition [3-5]. Therefore, in addition to dealing with all these problems, a patient-centered medical approach, along with effective self-management, plays a vital role for long-term transplant survivors for a better quality of life and health status [6].

Several factors can be described affecting the ability of the patient to follow recommendations and adhere to treatment, one of which is the health locus of control. Health locus of control, developed from social-learning theory, is considered a useful outcome for patient compliance and disease management and refers to the degree of control that individuals believe they possess over their personal health [7]. Health locus of control has drawn little attention in the field of solid organ transplantation in spite of a large body of literature regarding other chronic conditions. The only data related to liver transplant recipients belongs to a recent study including 222 individuals after heart, kidney, lung, and liver transplantation who scored highest in the powerful others subscale of health locus of control [8]. However, the study population included only two liver transplant recipients. In spite of health locus of control studies including mainly kidney, lung, and heart transplants, it remains unclear both how liver transplant recipients dominate their personal health status and to what extent it is associated with health behaviors. Therefore, the aim of this prospective study was to investigate health locus of control and physical activity, one of the main components of health behavior, and their relationship in a sample of liver transplant outpatients, a group that does not appear to be specifically studied in health locus of control literature. It was hypothesized that greater endorsement of physical activity would be associated with higher levels of internal health locus of control in liver transplant recipients.

## Materials and methods

This cross-sectional study included liver transplant recipients who underwent liver transplantation at least three months prior to the study initiation. The study was conducted in accordance with the ethical standards of the Helsinki Declaration and was approved by the Institutional Non-invasive Research Ethics Board (protocol number: 4839-GOA, approval number: 2019/18-23). All subjects gave written consent to participate in the study after receiving appropriate verbal and written information.

Subjects were invited to participate in the study during their regular visit to the liver transplant outpatient clinic in the Department of General Surgery, Dokuz Eylul University, between July 2019 and August 2020. Inclusion criteria were as follows: age between 18 and 65 years and Mini-Mental State Examination test score of ≥24. Subjects were excluded in case of multi-organ transplantation, previous orthopedic/musculoskeletal problems and/or other chronic diseases that could restrict physical activity, and severe co-morbidities (cardiovascular, neurologic, vestibular, psychiatric, etc.). During the period of data collection, 146 patients were invited to the study, 76 of whom had accepted to participate. Seventy patients were excluded as they did not meet the inclusion criteria or had not given informed consent.

Data collection

The Turkish version of the Multidimensional Health Locus of Control - Form C was used to measure the beliefs about control of one’s illness and the determinants of the existing medical condition such as the actions of himself/herself, chance, doctors, and other people [9]. Multidimensional Health Locus of Control - Form C consists of 18 items responded to on a 6-point Likert scale (1=strongly disagree to 6=strongly agree) and contains four sub-dimensions (Appendix 1) [9]: (1) Internal: it measures an individual’s tendency to believe that health outcomes are due mainly to one’s own behavior including six items. (2) Chance: it measures an individual’s tendency to believe that health outcomes are due mainly to chance, luck, or fate including six items. (3) Doctors: it measures an individual’s tendency to believe that health outcomes are due mainly to doctors and other health professionals, including three items. (4) Powerful others: it measures an individual’s tendency to believe that health outcomes are due mainly to other people, including three items. The scores range from 6 to 36 for internal and chance dimensions, whereas it ranges from 3 to 18 for doctors and powerful others dimensions. A higher score indicates a stronger belief about the influence of a given factor on health outcomes. Internal health locus of control is classified as internal belief, while the other three dimensions as external belief. Cronbach’s alpha was found to be 0.70 and more for the original questionnaire subscales [10]. For the Turkish version, Cronbach’s alpha was 0.82 for internal, 0.88 for chance, 0.63 for doctors, and 0.57 for powerful others subscales, and the test-retest reliability was 0.88 for internal, 0.83 for chance, 0.72 for doctors, and 0.57 for powerful others subscales [9].

The Turkish version of the International Physical Activity Questionnaire - Short Form was used to assess the physical activity level of the subjects [11]. The International Physical Activity Questionnaire - Short Form is a reliable and valid tool to screen physical activity levels over the preceding seven days. It consists of four sections and seven questions in total. It provides information on sitting, walking, moderate-intensity, and vigorous-intensity activities. The total score is calculated by multiplying the duration and frequency of activity by the value of a metabolic equivalent (MET) at activities of each different intensities (8 MET for vigorous-intensity activity, 4 MET for moderate-intensity activity, and 3.3 MET for walking), and a “MET-minute/week” score is obtained. Physical activity levels are classified according to the total score as low (<600 MET-min/week), moderate (600-3,000 MET-min/week), and high (>3,000 MET-min/week).

Data analysis

Statistical analysis was performed using Statistical Product and Service Solutions (SPSS, version 24; IBM SPSS Statistics for Windows, Armonk, NY). Visual (histogram and probability graphs) and analytical methods (Shapiro-Wilk test) were used to analyze the normality of data distribution. According to this test, most of the variables were not normally distributed. Therefore, non-parametric tests were used for statistical analysis. Continuous variables were expressed as medians and interquartile ranges (25th-75th percentile), while categorical variables were expressed as numbers (n) and percentages (%). The Spearman correlation coefficient was used to investigate the relationship between physical activity parameters and the health locus of control subscales. The strength of correlations was classified as very weak (r=0-0.19), weak (r=0.2-0.39), moderate (r=0.40-0.59), strong (r=0.6-0.79), and very strong (r=0.8-1). Health locus of control of liver transplant recipients with different physical activity levels (low and moderate) were compared with the Mann-Whitney U test. The statistical significance level was set at p<0.05. The effect size was calculated using Cohen’s d, interpreted as small (d=0.2), medium (d=0.5), and large (d=0.8) based on benchmarks suggested by Cohen [12].

## Results

Seventy-six liver transplant recipients participated in this cross-sectional study. Table 1 includes the demographic and clinical characteristics of the participants, while Table 2 shows the health locus of control and physical activity level. Median scores for internal, chance, doctors, and powerful others health locus of control were 28.54 (6-36), 22.71 (6-36), 17.11 (3-18), and 12.53 (3-18), respectively. According to the maximum scores available for each subscale, liver transplant recipients in this current study scored 79% for internal, 63% for chance, 95% for doctors, and 70% for powerful others health locus of control. Table 3 shows inter-correlations between health locus of control subscale scores.

**Table 1:** Demographic and clinical characteristics of liver transplant recipients *Other (autoimmune hepatitis, primary biliary cirrhosis, primary sclerosing cholangitis, cyst hydatic, biliary atresia, subfulminant hepatitis). Values are expressed as median and interquartile ranges (25^th^-75^th^) or the number of individuals (%).

Variable	Result
Age (years)	48.37 (41.25-58)
Gender (n, %)	
Female	24 (31.6)
Male	52 (68.4)
Body mass index (kg/m²)	27.09 (23.82-29.41)
Etiology of pre-transplant liver disease (n, %)	
Viral hepatitis (B + C)	22 (28.9)
Viral hepatitis + Hepatocellular carcinoma	14 (18.4)
Cryptogenic liver cirrhosis	10 (13.2)
Alcoholic liver disease	8 (10.5)
Wilson’s disease	6 (7.9)
*Other	16 (21.1)
Donor type (n, %)	
Cadaveric	35 (46.1)
Living	41 (53.9)
Time passed after liver transplantation	
< 5 years	22 (28.9)
5-15 years	50 (65.8)
> 15 years	4 (5.3)

**Table 2:** Health locus of control and physical activity scores of liver transplant recipients MET: Metabolic Equivalent of Task. Values expressed as median and interquartile ranges (25th-75th)

Scores	Median (25th-75th)
Internal (6–36)	28.54 (23.25-34)
Chance (6–36)	22.71 (16-30)
Doctors (3–18)	17.11 (17-18)
Powerful others (3–18)	12.53 (10-16)
Moderate-intensity activity (MET)	205.60 (0-80)
Vigorous-intensity activity (MET)	129.73 (0-0)
Walking (MET)	711.89 (160.87-820.87)
Total score (MET-min/week)	980.31 (297-1287)
Sitting duration (hour)	7.38 (6-9)
Level of physical activity	n (%)
Low (<600 MET-min/week)	36 (47.4)
Moderate (600-3000 MET-min/week)	35 (46.1)
High (>3000 MET-min/week)	5 (6.6)

**Table 3:** Intercorrelations between HLC subscales (n=76) HLC: Health Locus of Control, *p<0.05, Spearman correlation coefficient

	Internal HLC		Chance HLC		Doctors HLC		Powerful others HLC	
	r	p	r	p	r	p	r	p
Internal HLC	-	-	0.25	0.02*	0.45	<0.001	0.43	<0.001
Chance HLC	0.25	0.02*	-	-	-0.05	0.63	0.45	<0.001
Doctors HLC	0.45	<0.001	-0.05	0.63	-	-	0.25	0.02*
Powerful others HLC	0.43	<0.001	0.45	<0.001	0.25	0.02*	-	-

When the participants were classified according to their total International Physical Activity Questionnaire - Short Form score, 36 subjects (47.4%) were determined to have low physical activity levels, while 35 subjects (46.1%) had moderate physical activity levels. Only five subjects (6.6%) had high physical activity levels.

**Figure 1:**
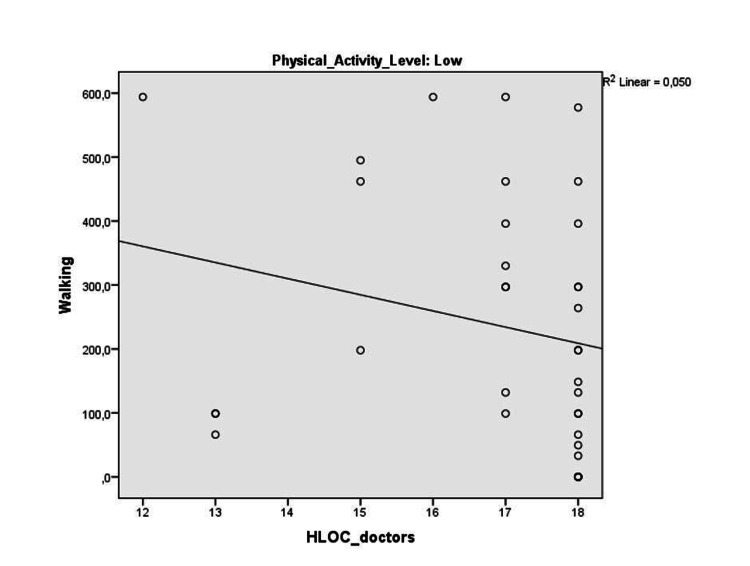
The correlation between HLOC_doctors and walking HLOC: Health Locus of Control

**Figure 2:**
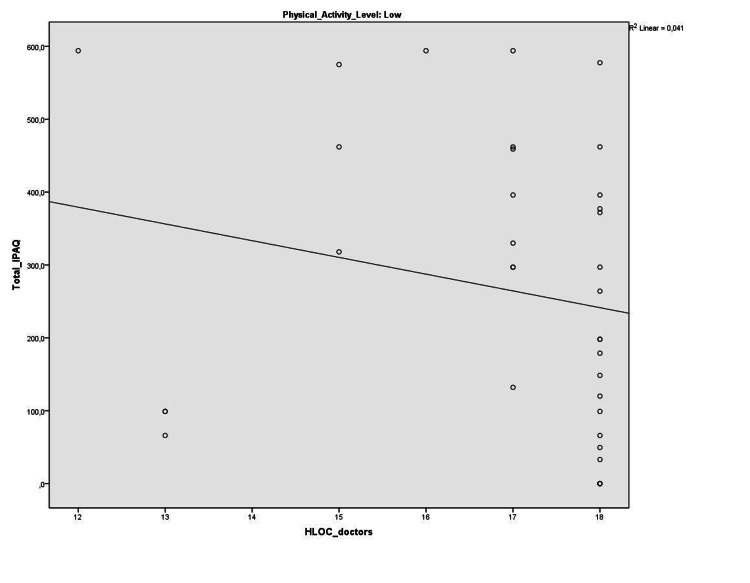
The correlation between HLOC_doctors and total IPAQ HLOC: Health Locus of Control, IPAQ: International Physical Activity Questionnaire

Within the whole subjects, no significant correlations were found between health locus of control subscale scores and physical activity parameters. Five liver transplant recipients with high physical activity levels were excluded from further analyses. We compared the health locus of control of our patients after physical activity level classification. Internal health locus of control was significantly higher in liver transplant recipients with moderate physical activity levels compared to liver transplant recipients with low physical activity levels (p=0.04) with a medium effect size (Cohen’s d=0.53) (Table 4). Within liver transplant recipients with low physical activity level, doctors sub-dimension of health locus of control was negatively correlated with total International Physical Activity Questionnaire - Short Form score and walking score (r=-0.37, p=0.02, and r=-0.38, p=0.02, respectively) (Figures 1-2).

**Table 4:** Comparison of health locus of control between LTRs with moderate and low physical activity levels MHLC: Multidimensional Health Locus of Control, LTRs: Liver Transplant Recipients, PA: Physical Activity, *p<0.05, d: Cohen’s d, Mann-Whitney U test. Values expressed as median and interquartile ranges (25^th^-75^th^)

MHLC-Form C sub-dimension scores	LTRs with low PA level	LTRs with moderate PA level	p	d
Internal (6–36)	28.00 (21.00-33.50)	31.00 (26.00-35.00)	0.04*	0.53
Chance (6–36)	23.50 (16.00-29.50)	22.00 (15.00-30.00)	0.92	-
Doctors (3–18)	18.00 (17.00-18.00)	18.00 (17.00-18.00)	0.54	-
Powerful others (3–18)	13.50 (8.00-16.00)	13.00 (11.00-16.00)	0.66	-

There was no correlation between health locus of control scores and physical activity parameters in liver transplant recipients with moderate physical activity levels.

## Discussion

This is the first study investigating the health locus of control and physical activity in a group of liver transplant recipients. We expected that liver transplant recipients who were physically active would present higher internal health locus of control, suggesting a possible interaction between the beliefs of one’s own control over disease management and health behavior. Our hypothesis was partially supported. The current study yielded three main findings. First, liver transplant recipients who participated in this study presented the highest beliefs regarding the influence of doctors on their health outcomes having a median score of 17.11 out of 18 (95%). Second, moderate physical activity was not satisfactory in our liver transplant recipients group as nearly half of the participants had low physical activity levels, which undermined the findings when analyzing this group in terms of health locus of control. Third, physical activity level showed a potential impact on the beliefs of liver transplant recipients’ own control over their disease state as internal health locus of control was higher in liver transplant recipients with moderate physical activity levels compared to those with low physical activity levels. Moreover, being the most influential dimension among the whole group, doctors' health locus of control was negatively associated with physical activity parameters in liver transplant recipients with low physical activity levels.

Health locus of control is a multidimensional concept by which individuals place their health outcomes in the control of their own behavior, defined as internal health locus of control, or in the control of powerful others or chance, defined as external health locus of control [13]. Internal health locus of control has been associated with successful treatment outcomes, increased patient compliance, and improved health behavior, including regular physical activity, weight loss, healthy diet, and reduced smoking and substance abuse in individuals with chronic diseases [14,15]. Perceived control is crucial to setting up effective management strategies for the disease and is related to increased motivation to change patients’ health behaviors. Health locus of control, which is important for adherence, self-management, and health behaviors, in chronic diseases has been previously evaluated among different solid organ transplants, but not in liver transplant recipients. Burker et al. [16] investigated pre-transplant health locus of control in lung transplantation candidates and found longer post-transplant survival in patients with medium to high levels of internal health locus of control compared to those with lower internal health locus of control [16]. Health locus of control was found to be one of the best predictors for overall health behavior in addition to an association with prophylaxis behavior among 107 heart transplant patients [3]. Internal health locus of control is also related to better quality of life and employment status in kidney transplant recipients [17].

Dabss et al. interviewed eight lung transplant recipients in order to describe their health locus of control and health behaviors and found a different typology referred to as dual health locus of control including both high internality and externality [18]. Our liver transplant recipients group manifested the highest ratio to maximum available score in terms of doctors' health locus of control, one of the subscales of externality, indicating our group’s tendency to believe that health outcomes were due mainly to doctors and other health professionals. We believe that sociocultural characteristics play an important role in the health locus of control and management of chronic diseases. In Turkish culture, doctors and other healthcare professionals have a considerable effect on patients’ perceptions of health. In the study of Buster et al., kidney transplant Turkish patients tend to believe in external forces in disease management [19]. As there is no study investigating Turkish liver transplant recipients’ health beliefs, we are unable to discuss our findings in terms of cultural aspects. Being a chronic condition and requiring a lifelong healthcare dependence, organ transplantation may lead the patients to rely mostly on doctors and healthcare professionals. Our results also showed a median internal health locus of control score reaching 79% of the maximum subscale score. Therefore, our findings also suggest a dual health locus of control in liver transplant recipients. Even though internal health locus of control has been associated with better health behaviors and more effective self-management in chronic diseases, dual health locus of control is suggested to be the most adaptive and even desirable response to the experience of chronic illness as it promotes better cooperation with the medical team [20]. Patients with breast cancer and diabetes relying on both high internality and externality believed that the best health management could be achieved by cooperation with clinicians [20,21]. Moreover, our findings indicated significant correlations between internal health locus of control scores and all external subscale scores. This finding was unusual, as typically there is no association between internality and externality. However, individuals with chronic illnesses commonly manifest a simultaneous endorsement of both health locus of control [20]. Similar scores for internal and powerful others' health locus of control may be attributable to high illness perception [13]. Parallel to other chronic conditions, organ transplantation probably leads to familiarization with the healthcare system and, thus, a complicated perception of health and illness from the point of a patient [18].

As the internal health locus of control had been associated with better health outcomes and health behaviors, we hypothesized to find a correlation between the internal health locus of control and physical activity. However, our liver transplant recipients group comprised patients with low and moderate physical activity levels, except for only five participants. Although we could not detect any significant relationship between health locus of control and physical activity among the whole group, liver transplant recipients with moderate physical activity levels manifested higher internal health locus of control compared to liver transplant recipients with low physical activity levels. This was the most important finding of this current study supporting our hypothesis. Therefore, we suppose that greater endorsement of physical activity would benefit individuals to take their own control over rather than chance, doctors, and other people. Moreover, this finding could be interpreted oppositely that liver transplant recipients with higher internal health locus of control could be more successful in achieving better physical activity levels. We believe that the bidirectional relevancy can be used for developing better strategies for health management after liver transplantation.

Our second finding supporting the main hypothesis includes a negative association between doctor health locus of control and walking and total scores of the International Physical Activity Questionnaire - Short Form in liver transplant recipients with low physical activity levels. As walking and physical activity levels increase, liver transplant recipients’ tendency to “believe health outcomes occur due mainly to doctors” decreases in this group. Therefore, we believe that promoting physical activity after liver transplantation will help develop better control that individuals believe they possess over their personal health. There is today available evidence that physically active behavior is an important predictor for the long-term health status of transplant recipients [2,4]. However, physical activity level is far from being ideal in liver transplant recipients similar to the findings of the current study [5,6]. The majority of our subjects (47.4%) engaged in low levels of physical activity. Barriers and motivators for physical activity after liver transplantation should be addressed in future studies.

The most important limitation of the current study is the lack of a comparison group such as healthy controls, patients with liver disease listed for transplant, or more interestingly other solid organ transplant recipients. Moreover, the range of time since transplantation was a little bit wide, which could possibly affect our main outcomes. We also could not explore the findings regarding liver transplant recipients with high physical activity levels as there were only five patients in our study sample. Therefore, we were only able to compare the health locus of control of liver transplant recipients with low and moderate physical activity levels. We also used a patient-centered, self-report measurement relying on the participants’ recall ability including a particular time (last seven days) to assess physical activity level. However, an objective method, such as an accelerometer, would provide more accurate data on physical activity parameters. Moreover, we believe that addressing health locus of control and physical activity levels and their association with a transplantation waiting list and after transplantation would be more beneficial to reflect the change over time and set up effective management strategies during the whole transplantation process.

## Conclusions

To the best of our knowledge, this is the first study exploring health beliefs and their association with health behavior in a group of liver transplant recipients. Our study population manifested a dual health locus of control, including both internality and externality, but slightly higher in favor of externality (higher degree for doctors' health locus of control). Liver transplant recipients with higher physical activity presented higher internal health locus of control. Although dual health locus of control is suggested to be the most adaptive and even desirable response to the experience of chronic illness as it promotes better cooperation with the medical team, promoting physical activity will help develop a higher internal health locus of control for better health management and health behavior after liver transplantation.

## Appendices

**Table 5:** The Multidimensional Health Locus of Control - Form C *Permission was obtained from the authors' of the Turkish version of the original questionnaire.

Internal (6 items)
If my condition worsens, it is my own behavior which determines how soon I feel better again
I am directly responsible for my condition getting better or worse
Whatever goes wrong with my condition is my own fault
The main thing which affects my condition is what I myself do
I deserve the credit when my condition improves and the blame when it gets worse
If my condition takes a turn for the worse, it is because I have not been taking proper care of myself
Chance (6 items)
As to my condition, what will be will be.
Most things that affect my condition happen to me by chance.
Luck plays a big part in determining how my condition improves
Whatever improvement occurs with my condition is largely a matter of good fortune.
If my condition worsens, it’s a matter of fate
If I am lucky. my condition will get better
Doctors (3 items)
If I see my doctor regularly, I am less likely to have problems with my condition
Whenever my condition worsens, I should consult a medically trained professional
Following doctor's orders to the letter is the best way to keep my condition from getting worse
Powerful others (3 items)
Other people play a big role in whether my condition improves. stays the same, or gets worse.
In order for my condition to improve, it is up to other people to see that the right things happen
The type of help I receive from other people determines how soon my condition improves

## References

[1] Dunn MA, Rogal SS, Duarte-Rojo A, Lai JC (2020). Physical function, physical activity, and quality of life after liver transplantation. Liver Transpl.

[2] Yıldız TA, Demir SG (2019). Expectations of liver transplantation candidates’ and their relatives and difficulties they experience. Cukurova Med J.

[3] Mierzyńska A, Kokoszka A, Jerzak-Wodzyńska G, Sobieszczańska-Małek M, Zieliński T, Piotrowicz R (2021). Involvement in health behavior after heart transplantation: the role of personal resources and health status. Single-center observational study. Front Psychol.

[4] Ng YH, Litvinovich I, Leyva Y (2022). Medication, healthcare follow-up, and lifestyle nonadherence: do they share the same risk factors?. Transplant Direct.

[5] Hreńczuk M, Bieniak A, Pazik J, Małkowski P (2018). Analysis of health behaviors in patients after liver transplant. Transplant Proc.

[6] Xing L, Chen QY, Li JN, Hu ZQ, Zhang Y, Tao R (2015). Self-management and self-efficacy status in liver recipients. Hepatobiliary Pancreat Dis Int.

[7] Marton G, Pizzoli SF, Vergani L (2021). Patients' health locus of control and preferences about the role that they want to play in the medical decision-making process. Psychol Health Med.

[8] Milaniak I, Dębska G, Król B, Wierzbicki K, Przybyłowski P (2022). Health locus of control and health behaviors in organ transplant recipients: a multicenter study. Transplant Proc.

[9] Krespi MR, Arslan I (2014). Multidimensional health locus of control scale C form: a reliability and validity study. JPCE.

[10] Wallston KA, Stein MJ, Smith CA (1994). Form C of the MHLC scales: a condition-specific measure of locus of control. J Pers Assess.

[11] Saglam M, Arikan H, Savci S, Inal-Ince D, Bosnak-Guclu M, Karabulut E, Tokgozoglu L (2010). International physical activity questionnaire: reliability and validity of the Turkish version. Percept Mot Skills.

[12] Lakens D (2013). Calculating and reporting effect sizes to facilitate cumulative science: a practical primer for t-tests and ANOVAs. Front Psychol.

[13] Kilic M, Arslan S (2021). Self-efficacy and its association with locus of control in diabetes in Turkey. Clin Exp Health Sci.

[14] Mercer DA, Ditto B, Lavoie KL, Campbell T, Arsenault A, Bacon SL (2018). Health locus of control is associated with physical activity and other health behaviors in cardiac patients. J Cardiopulm Rehabil Prev.

[15] Dogonchi M, Mohammadzadeh F, Moshki M (2022). Investigating the relationship between health locus of control and health behaviors: a systematic review. Open Public Health Journal.

[16] Burker EJ, Evon DM, Galanko J, Egan T (2005). Health locus of control predicts survival after lung transplant. J Health Psychol.

[17] Grubman-Nowak M, Dębska-Ślizień A, Renke M (2022). Employment after renal transplantation vs. the health locus of control and the quality of life. Int J Occup Med Environ Health.

[18] Dabbs ADV, Kim Y, Hamdan-Mansour A, Thibodeau A, Curry KM (2006). Health locus of control after lung transplantation: implications for managing health. J Clin Psychol Med Settings.

[19] Buster S, Ozsaker E (2022). Locus of control, self-efficacy perception and treatment adherence in kidney transplant patients. Transpl Immunol.

[20] Wallston KA, Wallston BS (1982). Who is responsible for your health? The construct of health locus of control. Social Psychology of Health and Illness.

[21] Helgeson VS (1992). Moderators of the relation between perceived control and adjustment to chronic illness. J Pers Soc Psychol.

